# Preventive effect and safety of Chinese herbal medicine mouthwash in chemotherapy-induced oral mucositis: A protocol for systematic review and meta analysis

**DOI:** 10.1097/MD.0000000000023387

**Published:** 2020-12-04

**Authors:** Jianfeng Zhang, Junfei Feng, Yunxia Liu

**Affiliations:** a Department of Oncology, Hangzhou Fuyang Hospital of Traditional Chinese Medicine; b Department of Oncology, The Third People's Hospital of Hangzhou, Hangzhou, Zhejiang, China.

**Keywords:** chemotherapy, Chinese herbal medicine, meta-analysis, mouthwash, oral mucositis, protocol, systematic review

## Abstract

**Background:**

Oral mucositis (OM), one of the most common side effects for cancer patients who have undergone chemotherapy, can cause severe impairment to patients’ functional ability and impact their quality of life, resulting in delayed and/or incomplete treatment. Traditional Chinese medicine offers empirical herbal decoctions to gargle for the prevention of chemotherapy-induced OM; however, evidence for its clinical efficacy and safety is lacking. Therefore, we provide a protocol to evaluate the preventive effect and safety of Chinese herbal medicine mouthwash in chemotherapy-induced OM.

**Methods:**

We will comprehensively retrieve relevant articles published till August 15, 2020, in the following electronic databases: the Cochrane Library, PubMed, EMBASE, Chinese National Knowledge Infrastructure Database, Chinese Biomedical Literature Database, Chinese Science and Technique Journals Database, and the Wan-fang Database. Only randomized controlled trials will be included. We will use the criteria provided by the Cochrane Handbook for quality assessment and risk assessment of the included studies and use the RevMan 5.3 software for meta-analysis of the preventive effect and safety.

**Results:**

This study will assess the preventive effect and safety of Chinese herbal medicine mouthwash in chemotherapy-induced OM.

**Conclusion:**

This systematic review will provide evidence-based medical corroboration for the clinical application of the Chinese herbal medicine mouthwash in chemotherapy-induced OM.

**PROSPERO registration number:**

CRD42020206614.

## 1 Introduction

Chemotherapy is one of the main therapeutic approaches for malignant tumors and often causes gastrointestinal reactions, myelosuppression, and many other adverse reactions.^[1]^ Oral mucositis (OM), one of the most frequent complications of chemotherapy in cancer patients, mainly manifests as erythematous and painful ulcerative lesions of the oral mucosa.^[2]^ According to previous studies, OM is reported in approximately 20% to 40% of patients receiving conventional chemotherapy. Furthermore, the incidence of this complication is up to 80% in patients receiving high-dose chemotherapy.^[3,4]^

Cancer patients with OM may experience dysphagia, alterations in taste, and secondary infections. As OM worsens, it complicates the treatment, influences the course of chemotherapy, extends hospitalization, and decreases the patient's quality of life.^[5]^ Owing to these serious clinical outcomes of OM, it may indirectly decrease the survival rate or dose reductions.^[6]^

Some preventive methods help decrease the incidence, severity, and duration of OM to different degrees, such as granulocyte colony-stimulating factor, keratinocyte growth factor, honey intake, and low-level laser therapy.^[7–9]^ However, the evidence-based clinical practice guidelines for the prevention of OM have not been released to date.^[10]^ Because of the limitations of efficacy from randomized controlled trials (RCTs) and the lack of substantial guidelines, it is advisable to attempt multitudinous preventive strategies for chemotherapy-induced OM. Chinese herbal medicine (CHM) decoction, which consists of only Chinese herbs, is used as a mouthwash for preventing the occurrence of OM in cancer patients and should be further studied as an option for alleviating this condition.^[11]^

OM caused by chemotherapy is also called “kou chuang” based on traditional Chinese medicine (TCM) theory. Over the past 2000 years, Chinese clinicians have shown good performance in treating OM.^[12]^ In the early years, Meyer-Hamme et al^[13]^ reported positive outcomes on the use of TCM-based interventions for the management of chemotherapy-induced OM among cancer patients worldwide. Recently, a number of CHM-based intervention clinical trials have been conducted. However, despite extensive clinical studies, evidence-based medical corroboration of traditional CHM decoction applied in patients with chemotherapy-induced OM is insufficient. Thus, adopting a meta-analysis approach to evaluate the preventive effect and safety of CHM mouthwash in chemotherapy-induced OM is essential.

## 2 Methods

### 2.1 Protocol and registration

The protocol of this study has been registered on the International Prospective Register of Systematic Review (PROSPERO). The registration number is CRD42020206614. We report the procedure of this protocol following the Preferred Reporting Items for Systematic Reviews and Meta-analyses guidelines.^[14]^

### 2.2 Inclusion criteria

#### 2.2.1 Type of studies

Only clinical RCTs of CHM mouthwash for chemotherapy-induced OM will be included. There is no restriction with respect to language or publication status.

#### 2.2.2 Type of participants

Cancer patients of any age diagnosed with OM after chemotherapy, with no reported abnormalities in the oral mucosa before chemotherapy, and with no regard to past surgical history, cancer type, chemotherapy regimens, sex, or race, will be included.

#### 2.2.3 Types of interventions

The patients in the study group will be instructed to gargle with CHM decoction before or on the day of chemotherapy. The control group will be treated with placebo, other standard agents, conventional western medicine, or no treatment. The methods, dosage, and course of treatment used in the control group are the same as those in the study group.

#### 2.2.4 Types of outcome measures

##### 2.2.4.1 Primary outcomes

The incidence of chemotherapy-induced OM and the incidence of severe OM (≥grade 3) according to the Common Terminology Criteria Adverse Events (CTCAE) or World Health Organization (WHO) criteria will be calculated.

##### 2.2.4.2 Secondary outcomes

The incidence of OM in each degree (grade 1, grade 2, grade 3, and grade 4) according to CTCAE or WHO criteria, oral mucosal pain according to visual analog scale, and the occurrence of adverse reactions will be calculated.

### 2.3 Exclusion criteria

Trials with one of the following conditions will be excluded:

1. Non-RCTs, quasi-RCTs, retrospective studies, case reports, and reviews;
2. OM caused by other reasons;
3. TCM mouthwash combined with other treatments, especially conventional western medicine;
4. Duplicated publications, the data cannot be synthesized, and the full text cannot be obtained.

### 2.4 Search strategy

We will search the following 7 electronic databases: the Cochrane Library, PubMed, EMBASE, Chinese National Knowledge Infrastructure Database, Chinese Biomedical Literature Database, Chinese Science and Technique Journals Database, and the Wan-fang Database. The limitations of the time interval are from the database inception to August 15, 2020. We will combine the subject word with a free word to acquire better retrieval results. Search terms are as follows: “oral mucositis,” “chemotherapy,” and “Chinese herbal medicine.” There are no language or publication restrictions. Meanwhile, the reference lists of included studies and gray literature will also be taken into consideration. The complete search strategy of PubMed is demonstrated in Table 1.

**Table 1:** Search strategy for the PubMed database.

Number	Entry terms
#1	Medicine, Chinese Traditional [MeSH]
#2	(Traditional Chinese Medicine) OR (Chung I Hsueh) OR (Hsueh, Chung I) OR (Traditional Medicine, Chinese) OR (Zhong Yi Xue) OR (Chinese Traditional Medicine) OR (Chinese Medicine, Traditional) OR (Traditional Tongue Diagnosis) OR (Tongue Diagnoses, Traditional) OR (Tongue Diagnosis, Traditional) OR (Traditional Tongue Diagnoses) OR (Traditional Tongue Assessment) OR (Tongue Assessment, Traditional) OR (Traditional Tongue Assessments)
#3	#1 OR #2
#4	Mouthwashes [MeSH]
#5	(Mouth Rinse) OR (Mouth Rinses) OR (Rinse, Mouth) OR (Rinses, Mouth) OR (Mouth Bath) OR (Bath, Mouth) OR (Baths, Mouth) OR (Mouth Baths) OR (Mouth Wash) OR (Wash, Mouth)
#6	#4 OR #5
#7	Drug Therapy [Mesh]
#8	(Therapy, Drug) OR (Drug Therapies) OR (Therapies, Drug) OR (Chemotherapy) OR (Chemotherapies) OR (Pharmacotherapy) OR (Pharmacotherapies)
#9	#7 OR #8
#10	Stomatitis [Mesh]
#11	(Stomatitides) OR (Oral Mucositis) OR (Mucositides, Oral) OR (Oral Mucositides) OR (Oromucositis) OR (Oromucositides) OR (Mucositis, Oral)
#12	#10 OR #11
#13	Oral Ulcer [Mesh]
#14	(Oral Ulcers)OR (Ulcer, Oral) OR (Ulcers, Oral) OR (Mouth Ulcer) OR (Mouth Ulcers) OR (Ulcer, Mouth) OR (Ulcers, Mouth)
#15	#13 OR #14
#16	#12 OR 15
#17	#9 and #16
#18	(Randomized controlled trial) [Publication Type] OR Randomized [Title/Abstract] OR Placebo [Title/Abstract]
#19	#3 and #6 and #17 and #18

### 2.5 Selection of studies

**Figure 1:**
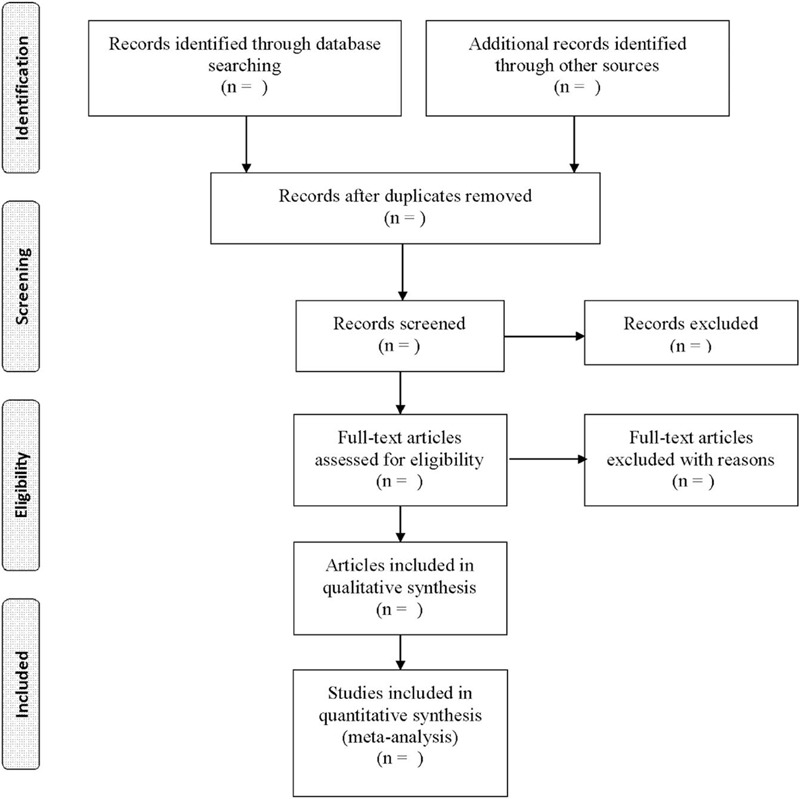
Process of study selection.

We will use Endnote X9 to manage all retrieved articles. First, all duplicated studies will be filtered by this software. Second, 2 researchers will independently browse the titles and abstracts of the remaining studies according to the inclusion criteria. Last, the full texts of all possibly relevant studies will be downloaded to allow further independent selection by the 2 reviewers. In addition, 2 reviewers will cross-check the included studies. A discussion with a third party will resolve any form of dispute. Figure 1 shows the study selection procedures.

### 2.6 Data extraction and management

Two investigators will separately extract the following data: general information of the included studies, such as randomized methods, random concealment, blindness, number of cases in each group, age, gender, condition, and diagnostic criteria; and the intervening measures and outcomes, such as the incidence of OM and adverse events.

### 2.7 Assessment of the risk of bias

Two researchers will independently evaluate the risk of bias for the included studies using the Cochrane Handbook for Systematic Reviews of Interventions tools. The following items will be assessed: random sequence generation, allocation concealment, blinding of participants and personnel, blinding of outcome assessment, incomplete outcome data, selective reporting, and other sources of bias. Each domain is considered as low (met all criteria), unclear (trials with insufficient information for judgment), or high risk (met none of the criteria) of bias. Discrepancies in the interpretation will be resolved by consensus or with the involvement of a third party.

### 2.8 Data analysis

We will use RevMan 5.3 software supported by the Cochrane collaboration to perform a meta-analysis. For dichotomous outcomes, we will use the relative risk to analyze. Conversely, for continuous outcomes, we will use the mean difference. The uncertainty is expressed with 95% confidence intervals, and the heterogeneity is measured using the I^2^ statistic. If there is no statistical heterogeneity among the studies (I^2^ < 50%, *P* > .1), a fixed-effects model will be used. Otherwise, a random-effects model will be applied. When meta-analysis is not available, a descriptive analysis will be performed.

#### 2.8.1 Subgroup analysis

Subgroup analyses based on the type of control group intervention (e.g., placebo, no treatment, conventional western medicine, or other standard agents), dose of chemotherapy, and chemotherapy regimens will be conducted to explore the sources of heterogeneity.

#### 2.8.2 Sensitivity analysis

We will remove the included studies one at a time and synthesize the remaining literature to evaluate the credibility of the synthetic outcomes.

#### 2.8.3 Publication bias

For outcomes, if 10 or more RCTs are included, we will assess the publication bias by the symmetry of the funnel plots.

### 2.9 Grading of Recommendations Assessment, Development, and Evaluation (GRADE)

We will use the GRADE to evaluate the quality of evidence of outcomes.^[15]^

### 2.10 Ethics and dissemination

No ethical approval is required as our study will be performed based on published articles. Furthermore, the findings of this systematic review will be published through peer-reviewed publications.

## 3 Discussion

In recent years, OM caused by chemotherapy has become a problem that cannot be ignored in the general management of cancer patients.^[16]^ Its pathogenesis may be explained by a 5-stage process. First, the toxicity of chemotherapy drugs destroys the cellular and generation of free radicals, resulting in the death of basal epithelial cells. Next, the increase in inflammatory factors exacerbates cell death. The upregulation of proinflammatory cytokines causes mucosal ulcerations, which accelerate a secondary infection. In the fifth stage, epithelial proliferation, as well as cellular and tissue differentiation, occurs.^[17–19]^ Clinicians have not reached a consensus on the principles of prevention and treatment for OM caused by chemotherapy, and still, there is no specific management standard.^[20]^

Based on the abovementioned progression of pathogenesis, oxidative stress and inflammation are related to the development of OM caused by chemotherapy. Many TCM decoctions that possess antioxidant and anti-inflammatory properties should take an effective part in preventing this complication.^[21]^ In fact, a variety of Chinese herbal medicines prevent or treat chemotherapy-induced OM among Chinese cancer patients, indicating the potential of interventions based on TCM for oral care among patients.^[22]^ Therefore, we will conduct this systematic review and meta-analysis to evaluate the preventive effect and safety of CHM mouthwash in chemotherapy-induced OM.

The results of this study can provide a possible consideration for CHM as a treatment for chemotherapy-induced OM. We hope that clinicians will acquire the basis for the prevention of OM with CHM and obtain the best choice for the management of patients according to our results. However, although a comprehensive search will be performed, we will search for papers published in only Chinese and English, which may result in bias.

## Author contributions

**Data curation:** jianfeng zhang, Junfei Feng.

**Funding acquisition:** Yunxia Liu.

**Methodology:** jianfeng zhang.

**Resources:** jianfeng zhang.

**Software:** jianfeng zhang.

**Supervision:** Yunxia Liu.

**Writing – original draft:** jianfeng zhang.
